# Structure learning in action

**DOI:** 10.1016/j.bbr.2009.08.031

**Published:** 2010-01-20

**Authors:** Daniel A. Braun, Carsten Mehring, Daniel M. Wolpert

**Affiliations:** a Computational and Biological Learning Lab, Department of Engineering, University of Cambridge, UK; b Bernstein Center for Computational Neuroscience, Freiburg, Germany

**Keywords:** Structure learning, Adaptive motor control, Learning-to-learn, Visuomotor learning, Dimensionality reduction, Variability

## Abstract

‘Learning to learn’ phenomena have been widely investigated in cognition, perception and more recently also in action. During concept learning tasks, for example, it has been suggested that characteristic features are abstracted from a set of examples with the consequence that learning of similar tasks is facilitated—a process termed ‘learning to learn’. From a computational point of view such an extraction of invariants can be regarded as learning of an underlying structure. Here we review the evidence for structure learning as a ‘learning to learn’ mechanism, especially in sensorimotor control where the motor system has to adapt to variable environments. We review studies demonstrating that common features of variable environments are extracted during sensorimotor learning and exploited for efficient adaptation in novel tasks. We conclude that structure learning plays a fundamental role in skill learning and may underlie the unsurpassed flexibility and adaptability of the motor system.

## 1 The structural learning hypothesis

**Fig. 1:**
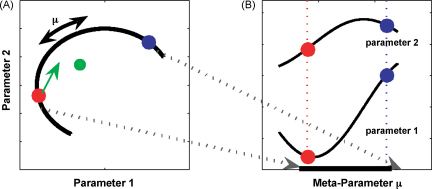
Schematic diagram of structural learning. (A) The task space is defined by two parameters, but for the given task only certain parameter combinations occur (black line). This relationship is indicated by the curved structure which can be parameterized by a one-dimensional meta-parameter *μ*. However, a parametric learner that is ignorant of the structure has to explore the full two-dimensional space when re-adjusting the parameter settings. (B) A structural learner, in contrast, takes the relationship between the parameters into account. By adjusting only the meta-parameter *μ* the learning problem is effectively one-dimensional. Reprinted with permission from [66].

Consider operating a machine with a hundred dials that you can turn, each of which controls a single parameter (Fig. 1A shows just two of the 100 parameters). The machine is performing well at a particular task (red setting) and you want to adjust it for a new task. How could you adjust the hundred dials to find the setting for the new task (blue setting)? This is the problem the nervous system faces during learning, except there are not 100 dials but often several hundreds (e.g. muscles) or millions (e.g. synaptic strengths). One solution is to use optimization techniques to adjust each parameter, thereby exploring the entire 100-dimensional space. However, through experience you might find that for a set of related tasks the final settings of the dials have a certain fixed (or probabilistic) and possibly non-linear relation (thick black line). This would allow us to build a new meta-dial (with setting *μ*) that controls and restricts all the other dials to move along a lower dimensional structure in parameter space (Fig. 1B). Therefore, when presented with a new task on the same structure, the search is restricted to a subspace of the full parameter space, and all you need to do is adjust a single meta-dial thereby speeding up learning. This is the essence of structural learning (which we use synonymous with structure learning).

In a biological organism, controlling the dials corresponds to controlling internal parameters that determine the way in which sensory inputs are transformed into motor outputs. Changing these internal parameters leads to changes in the input–output mapping—that is learning [1]. From a theoretical point of view, the problems of how to construct the meta-dial and how to adjust it correspond to ‘structural learning’ and ‘parametric learning’ respectively. In structural learning, what is learned is the general form of the rules that govern a set of tasks, whereas parametric learning involves selecting the particular mapping that governs the current task.

Structural learning [2–8] essentially reduces the dimensionality of the space that the learning organism has to search to adapt to novel tasks. Such dimensionality reduction can be regarded as an extraction of invariants between different input–output mappings. Extracting such invariants and reducing the dimensionality of the search space will improve the efficiency of any learning algorithm [9] leading to faster learning for problems sharing a similar structure. Thus, structural learning could provide a mechanism for ‘learning to learn’ and transfer between tasks with the same task structure. While structural learning is expected to speed-up learning, not all ‘learning to learn’ effects require structural learning. For example, non-specifically increasing the rate at which all parameters change could speed-up learning but would not be specific to a particular set of tasks [10]. In the following we will focus on ‘learning to learn’ phenomena induced by structure learning.

## 2 Structure learning in animal cognition

Some of the earliest accounts of facilitated transfer between tasks with a similar structure were in the cognitive domain where monkeys had to choose one of two stimulus-objects (e.g. a cube or a cylinder), only one of which would lead to a reward. The same pair of stimulus-objects was repeatedly presented presented in randomized locations (i.e. left or right) for a block of trials. At the start of a new block two new stimulus-objects were used. Initially, the monkeys took many trials to learn the correct response, but in later blocks Harlow observed a greater than 95% success rate on the second trial of each block [11]. This suggests that the animals had learned the structure of the task that is the abstract rule that one stimulus receives a reward and the other does not. In this task ‘parametric learning’ within each block corresponds to identifying which stimulus is rewarded. The speed-up in learning over the blocks arises through the learning of the structure and the rapid increase in success rate within the first few trials of a block arises through parametric adaptation. Such a speed-up in learning was termed ‘learning to learn’ and now has a long tradition in experimental psychology.

Harlow noted that most animal experiments had studied learning as an isolated episode on a single task and, therefore, did not reflect learning in everyday life which involves experience over many tasks [12]. According to Harlow's hypothesis, animals learn slowly by trial and error when they are faced with a novel task but once they have had experience with a set of related tasks they can generalize to solve novel tasks within this class, leading to apparently insightful behaviour. Harlow defined this ‘learning to learn’ or ‘meta-learning’ as the formation of a ‘learning set’. Thus, the ‘learning set’ hypothesis of ‘learning to learn’ *“describes the ability of animals to slowly learn a general rule which can then be applied in order to rapidly solve new problem sets”* [13]. Historically, Harlow used the term ‘learning sets’ as a concept to bridge the gap between the behaviourists’ theory of learning by trial and error and the more insightful and discontinuous forms of learning suggested by Gestalt psychologists [13,14]. The concept of ‘learning sets’ is still employed in contemporary studies on animal learning, e.g. [15,16].

Further investigations showed how the features of the training sets determined learning rates. Substantial facilitation was reported for *intra-dimensional* shifts compared to *extra-dimensional* shifts in learning problems [17–20]. For example, pigeons exposed to coloured shapes, in which the colour was predictive of the reward, rapidly adjusted to novel colours (intra-dimensional shift) but not when the shape became predictive of the reward (extra-dimensional shift) [17,21]. The ability to solve intra-dimensional problems more quickly has been interpreted as learning of abstract dimensions such as colour or shape rather than particular physical instantiations like red or triangular.

Animals have also been shown to learn more complex rules by mastering conditional discriminations that depend on the presence of contextual stimuli [21–25]. For example, chimpanzees can be trained to discriminate between stimuli based on shape if the stimuli are presented on white background and by colour if the stimuli are presented on a black background [13]. In categorisation tasks animals have learnt discrimination rules that depend on abstract concepts like ‘same’ or ‘different’ [26–28], ‘triangularity’ [29], ‘symmetry’ [30] or ‘people’ versus ‘no-people’ [31] and ‘food’ versus ‘non-food’ [32–34]. Apes have even been reported to exploit abstract concepts for analogical reasoning [35].

## 3 Structure learning in cognitive neuroscience

**Fig. 2:**
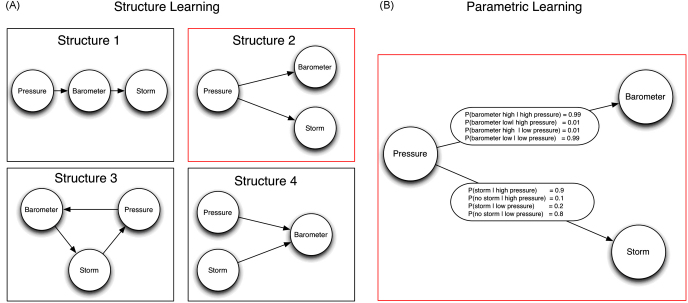
Example of a causal Bayesian network. (A) Four possible structures. The arrows represent the causal structure of the three variables pressure, barometer and storm that are represented by nodes. Structural learning is determining which of the possible structures is the best model of the data. In this case the readings of the barometer and the probability of a storm occurring are correlated but independent when conditioned on the variable pressure suggesting structure 2. (B) Parametric learning involves specifying the probability distribution that quantifies the strength of the causal connections given a particular structure. In this case there is a 0.01 probability of the barometer being broken and giving a false reading.

‘Learning to learn’ has also been reported in both children and adults [36–39]. However, only very few studies have related ‘learning to learn’ effects to the structure of the tasks. For example, in one study, participants were exposed to pairs of symbols one of which represented an initial state and the other an operator on that state which determined a third symbol (the final state). Subjects were then required to predict end-states given the initial-state and operator [39]. The participants showed transfer to problems with the same structure thereby demonstrating that structural coherence had a powerful effect on learning. Similarly, structural learning has been used to explain how humans generalize based on limited information, for example, when judging similarity between animals or numbers [40–42]. In addition, there have been investigations of structural learning in perceptual tasks [43]. However, the majority of studies of structural learning in cognitive science have not focussed on the issue of speeding up learning for novel tasks, but on the inference of causal structures between different observed variables [44–48]. For example, if we observe the three variables of pressure, barometer reading and the occurrence of storms, there is a strong correlation between the readings of the barometer and the probability of a storm occurring. However, this statistical co-variation does not imply a causal relationship (i.e. the barometer causing a storm). Mathematically, causal relationships are typically represented by graphical models [2,49] such as a directed graph consisting of nodes that represent the variables of interest (e.g. pressure, barometer and storm in Fig. 2A) and arrows that represent which variables have a causal influence on other variables and which variables are causally independent (e.g. both barometer and storm are independent conditional on pressure). Thus, there are two things that need to be learned in causal induction: (1) the causal structure (i.e. which variables are connected by arrows—Fig. 2A shows 4 possible structures) and (2) the strength of causal relations (e.g. what is the probability of a storm occurring given that the pressure is high? Fig. 2B shows a possible parameterisation given a particular structure). The first problem is the problem of structure learning, the second problem is the problem of learning the parameters of the structural model. In a number of experiments it has been found that humans have great difficulty inferring the causal structure of a set of variables from correlational data alone [47,50,51]. Conditional independence relations are hard to find because even very simple cases require subjects to track concurrent changes in several variables—e.g. three variables in the example above. The number of possible dependencies expands rapidly with the number of variables involved. This leads to a substantial computational load and even the most sophisticated statistical methods do not guarantee that a unique causal structure can be found. This is because when making inferences from correlational data, there will usually be several ‘Markov equivalent’ structures that explain the co-variations in the data equally well [49]. It is therefore not surprising that humans have been shown to use additional cues to identify causal dependencies like temporal order [52] and active interventions [47,50,51]—for example, by manipulating the barometer and testing whether this has an influence on the probability of a storm occurring. Structural learning and uncertainty over different structures have even been reported to explain human decision making in more complex sequential decision tasks that have previously been interpreted as exposing suboptimal behaviour on the part of the human decision makers [53].

## 4 Structural learning in motor control

In sensorimotor control it is natural to think of regularities between motor output and sensory inputs. These are imposed by both the physical properties of the body and the regularities in the environment or set of task to be performed. Structural learning could exploit these regularities to facilitate learning.

### 4.1 Adaptive control theory

Adaptive control is a branch of control theory aimed at problems in which some of the properties of the system that we wish to control are unknown. This approach uses two levels of analysis: ‘structural adaptive control’ and ‘parametric adaptive control’ [4,54]. In *parametric* adaptive control, knowledge of the structure of the control task is presupposed (e.g. the form of the equations of motion governing the dynamics of the arm) and only unknown parameters of the structure that are currently in play have to be estimated (e.g. the mass of a hand-held object). Parametric adaptive control, therefore, consists of estimating the parameters of the system (using one of a number of optimization procedures) and then using these parameter estimates in the control process. However, when the structural form itself is unknown, adaptive control relies on *structural* learning to develop a representation of the control process. Such structural learning, in its most general form, needs to determine the relevant inputs and outputs of the system, the dimensionality and form of the control problem, the relation between the control variables, their range of potential values and the noise properties associated with them. The theoretical literature has primarily investigated parametric adaptive control as this allows, by design, the adaptive control problem to be turned into a parametric optimization problem for which established system identification techniques can be employed to determine the parameters of the fixed model structure—see, for example [4,54]. The structural learning problem, while potentially the more powerful framework, is more difficult to handle. The difficultly arises because the relevant structure of the controller has to be determined, for which there are no standard techniques in adaptive control, before parametric identification can take place.

One control theoretic framework to study adaptive control is optimal feedback control theory in which the command, *u*, is driven as a function of sensory input, *x*, and the parameters of the system, *μ* such that *u* *=* *f*(*x,μ*) [65]. Here *f*() represents the structure of the task and *μ* the relevant parameters which vary across tasks. Critically, the representation of *μ*, and hence *f*(), depends on the task. For example, if across tasks the mass of a hand-held object varies then *μ* might represent the mass, whereas if the task involved wielding sticks of different lengths *μ* might represent the length of the stick. In adaptive control theory *u* *=* *f*(*x*,*μ*) is called a *parametric* adaptive control law, because it encapsulates the knowledge of how the unknown parameters *μ* are structurally related to the other control variables. Such parametric control laws can be used to study movement generation within the framework of optimal feedback control, and have been successful in explaining a wide range of human motor behaviour [55–61].

### 4.2 Evidence for structural learning in motor control

‘Learning to learn’ phenomena have also been observed in motor control tasks [10,62–72]. In [67], for example, human participants adapted and readapted to a sequence of repeating visuomotor displacements; that is they experienced blocks of trials with opposing (leftward and rightward) prismatic rotations. Not only did these participants show faster adaptation rates over the course of the blocks for both prisms (termed ‘dual adaptation’), but also when faced with a new larger prismatic displacement, participants showed facilitation of learning (‘adaptive generalization’ or ‘learning to learn’). Such ‘adaptive generalization’ has also been observed in a study which manipulated both the type of visual distortion and the type of task [72]. Subjects either walked on a treadmill or performed a balancing task. Within each of these tasks, subjects either wore a variety of distorting lenses or a single distorting lens. After training they were tested on a walking task that required obstacle avoidance. The authors found facilitation for this new task for subjects who had experienced the variety of lenses while walking on the treadmill, but no facilitation for those who had done the balancing task. This work adds to earlier studies by showing that task similarity (locomotion in this study) is critical for transfer [71] and that variability during practice facilitates retention and transfer [73].

The importance of variability during training is also emphasized in another visuomotor displacement study [70] showing that variable practice with different lenses (magnifying, minifying and up/down reversal) over the course of three weeks increased subjects’ adaptability to a novel visuomotor displacement compared to subjects who underwent training with only one set of magnifying lenses or sham lenses. Such non-specific speed-up of learning was found, for example, in [10], where subjects were trained on one motor learning task and transfer to another task assessed. This study demonstrated enhancements of learning and plasticity through facilitated transfer between motor tasks that share little structural similarity, such as from visuomotor rotations to sequence learning. Such non-specific enhancement of learning relies on different mechanisms from structural learning. Structural learning predicts facilitation effects that are specific for the sensorimotor structure that has been learned.

**Fig. 3:**
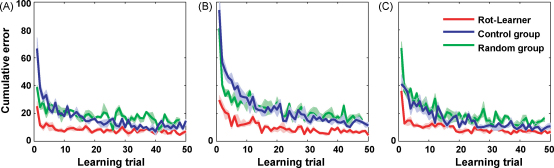
Structural learning of visuomotor rotations. (A) Learning curves for a block of +60° rotation trials performed by a group that had experienced random rotations before (R-learner, red), a control group that had only experienced movements with veridical feedback (blue) and a group that experienced random linear transforms and ±60° rotations (green). The rotation group shows strong facilitation. (B) Learning curves for a subsequent block of −60° rotation trials performed by the same groups. The interference effect that can be seen in the control group is strongly reduced in the rotation group. (C) Learning curves for a subsequent block of +60° rotation trials performed by the same groups. Again the random rotation group shows a performance advantage in the first 10 trials. The median error over all subjects is shown and the pertinent interquartile confidence interval. Reprinted with permission from [66] (For interpretation of the references to colour in this figure legend, the reader is referred to the web version of the article.).

While the motor studies described above have investigated ‘learning to learn’ effects, they have not directly tested structural learning as a basis for facilitation of learning. Recently, we have conducted a series of studies to directly test the idea of structural learning in motor control. In our tasks subjects had to learn visuomotor transformations between their actual and perceived hand movement in virtual reality reaching tasks—for example, a visuomotor rotation would induce the perceived hand movement to be a rotated version of the actual hand movement. Such visuomotor transformations can be chosen to have a particular structure (e.g. rotations, shearings or scalings) that requires the setting of specific parameters to be fully determined (e.g. rotation angle, shearing parameter or scaling factor). By randomizing these parameter settings in our experiment we could test how random experience of a particular structure affects learning of a new visuomotor transformation. In [66], for example, subjects experienced extended training either with random visuomotor rotations or with highly variable random linear transforms composed of rotations, shearings and scalings (Fig. 3). After extensive experience with these transformations we exposed both groups to a new fixed visuomotor rotation. The structural learning hypothesis predicts that subjects who had experienced random visuomotor rotations should exhibit strong facilitation when faced with a particular instance of this class of visuomotor transformation as was observed in our experiment. In contrast, the random transformation group performed no better than a naïve group, although they had extensive experience with rotations. The reason for this lack of transfer is that the random transformation group experienced rotations as part of a much less constrained structure of random linear transformations which did not allow the detection of a lower dimensional structure. Transfer between two blocks of visuomotor rotations with different rotation angles has been previously reported [74]—e.g. learning a 45° rotation facilitates learning a 90° rotation. However, previous studies did not provide any direct evidence for the importance of variable experience of a particular task structure (e.g. rotations) as opposed to experience with another task structure (e.g. linear transforms).

**Fig. 4:**
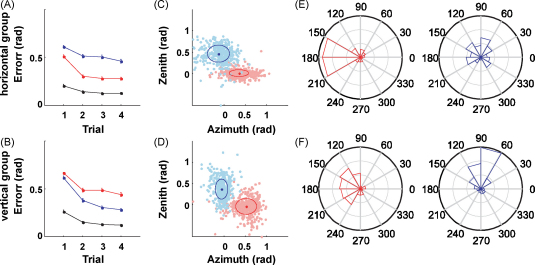
Structural learning of 3D rotations. (A) Angular error in probe blocks of horizontal (red) and vertical (blue) 45°-rotations experienced by a group that experienced random horizontal rotations before. There is a clear facilitation for learning the horizontal rotation. The black line indicates performance in the block of null-rotation (washout) trials preceding the probe block. (B) Performance error in the same probe blocks for a group that experienced random vertical rotations before. The facilitation pattern is reversed. (C and D) Movement variance shortly before trial end for both kinds of probe blocks. The variance in the task-irrelevant direction — perpendicular to the displacement direction — is significantly reduced for isostructural probe blocks (ellipses show standard deviation). This suggests that subjects explored less outside the structure they had learned during the random rotation blocks. (E and F) Circular histograms of initial movement adaptation from the 1st trial of the probe block to the 2nd trial. Subjects responded to probe blocks from the same structure in a consistent way correcting towards the required target. In contrast, in case of probe trials for a different structure, subjects also showed components of learning in the direction of the previously learned structure. Reprinted with permission from [66] (For interpretation of the references to colour in this figure legend, the reader is referred to the web version of the article.).

The structural learning hypothesis also predicts that, when learning a new transformation, the motor controller should, at least initially, explore preferentially along the structure (thick black line in Fig. 1A) and reduce deviations from the structure. To test this prediction, while subjects made reaching movements in three-dimensional space we exposed them to randomly varying rotations either around the vertical or the horizontal axis. Therefore each group of subjects experienced either a vertical or horizontal rotation structure, parameterized with a single rotation parameter [66]. Later in the experiment, vertical and horizontal rotation probe trials were introduced for both groups. Interestingly, both groups reacted very differently to the same probe trials. They showed structure-specific facilitation, variability patterns and exploration strategies (Fig. 4). Their endpoint variance was markedly reduced in the direction orthogonal to their learned structure. This suggests that exploration occurred preferentially along the previously learned structure and deviation from the structure was reduced. Reduction of variance has long been recognized as an important feature of learning [75]. Previously, it has been proposed that motor variance is reduced in the degrees of freedom that interfere with task goals, whereas variability in task-irrelevant dimensions is tolerated as a redundancy [55,76–80]. Structure learning is entirely compatible with this concept of redundancy in task-irrelevant dimensions, since the subspace found after learning might still comprise many redundant dimensions.

Previously, it has also been suggested that co-variation between different control variables can be exploited to reduce task variance [81–86], for example, when deviations of two variables compensate for each other, as has been observed for variations in body and pistol angles compensating for each other to achieve a steady pointing position [81,82]. The exploitation of such co-variations is consistent with structural learning and its effect on the structure of movement variability. Similar correlations between control variables have also been previously reported in the context of motor synergies [87–89] and generalized motor programmes [90,91]. Both concepts can be considered as structures that are scaled by “activation levels” and are therefore also compatible with the notion of structure learning.

**Fig. 5:**
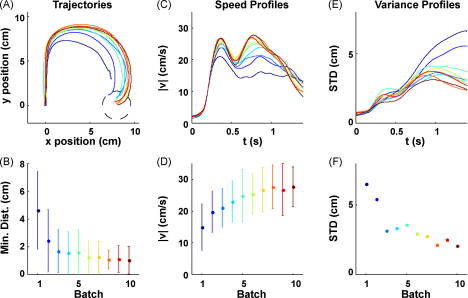
Evolution of within-trial adaptive behaviour for random rotation trials. (A) Mean hand trajectories for ±90° rotation trials in the first 10 batches averaged over trials and subjects (each batch consisted of 200 trials, approximately 5% of which were ±90° rotation trials). The −90° rotation trials have been mirrored about the *y* axis to allow averaging. Dark blue colours indicate early batches, green colours intermediate batches, red colours indicate later batches. (B) The minimum distance to the target averaged for the same trials as A (error bars indicate standard deviation over all trajectories and all subjects). This shows that subjects’ performance improves over batches. (C) Mean speed profiles for ±90° rotations of the same batches. In early batches, movements are comparatively slow and online adaptation is reflected in a second peak of the speed profile which is initially noisy and unstructured. (D) The magnitude of the second peak increases over batches (same format as B). (E) Standard deviation profiles for ±90° rotation trajectories computed for each trial batch. (F) Standard deviation of the last 500 ms of movement. Over consecutive batches the variability is reduced in the second part of the movement. Reprinted with permission from [65] (For interpretation of the references to colour in this figure legend, the reader is referred to the web version of the article.).

Once the structure of a control task has been learned, one can employ adaptive control methods to model adaptive motor responses. Consider, for example, picking up an empty milk carton that you believe to be full. Not only do you need to adapt your estimate of the carton's weight, but simultaneously you must exert control to move the carton to a desired location. We have shown that training on such unpredictable tasks leads to the formation of structure-specific adaptation strategies that can be understood within the framework of parametric adaptive optimal feedback control [65]. In such an adaptive optimal feedback controller the structure of the control task is represented by an adaptive internal model of the dynamics of the environment [92–94] and the parameter of that structure identifies all the environments belonging to the class that the internal model is suited for—e.g. an internal model for the manipulation of the milk carton that can be scaled according to its weight. In our experiments [65], we tested directly whether the behaviour can be modelled by adaptive optimal control principles. Subjects were exposed to unexpected visuomotor rotations to which they had to adapt while reaching. Importantly, the visuomotor rotations could not be predicted in any single trial and required ‘online adaptation’, since the mapping from hand to cursor changed all the time. We found clear signs of improvements in subjects’ adaptive behaviour over the course of many trials (Fig. 5). The adaptive control model could reproduce the stereotyped adaptive arm movements generated by subjects at the end of this ‘learning how to adapt’ process by assuming an internal model that knows about the visuomotor rotation structure and tries to adapt the particular rotation angle parameter in each trial. Interestingly, this model predicts that there should be no adaptation process for visuomotor perturbations that do not change the mapping between inputs and outputs, for example when the target jumps. In accord with these predictions we found that another group of subjects who experienced random target jumps showed no learning-to-learn over the course of trials [65]. This suggests that subjects who experienced random rotations had learned an adaptation strategy that was specific for the structure of the environmental variability they encountered during training.

## 5 A Bayesian perspective

**Fig. 6:**
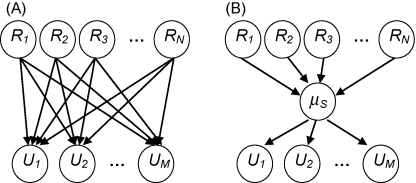
Structural learning in Bayesian networks. (A) The nodes of the Bayesian Network represent random variables such as sensory inputs *R*_*j*_ and motor outputs *U*_*k*_. The arrows indicate causal dependencies that are usually expressed via parameterized probability density functions. Learning the parameters of the full joint probability distribution in this network will require substantial computations. (B) In this network there is a hidden variable *μ* that corresponds to what we have called a ‘meta-parameter’. The joint probability distribution over all variables splits up into a product of conditional distributions with regard to *μ*. This substantially reduces the dimensionality of the parameter space. In our experiments *μ* corresponds for instance to internal variables specific for rotations. Reprinted with permission from [66].

Structural learning can also be considered from a Bayesian point of view, in which the learner maintains a probability distribution over possible structures that could explain the data. Such structural learning is typically studied in the framework of Bayesian networks (Fig. 6). Ultimately, a Bayesian network is a graphical method to efficiently represent the joint distribution of a set of random variables [2,6,8]. In cognitive science, for example, Bayesian graphical networks are widely used to study structure learning in causal induction [95,96]. In the case of sensorimotor learning, we can consider a Bayesian network in which *N* random variables represent the receptor input $R_{1},R_{2},...,R_{N}$ (e.g. retinal input, proprioceptive input, or later stages of neural processing) and *M* variables the motor output $U_{1},U_{2},...,U_{M}$ (e.g. muscle activations or earlier stages of neural processing) (Fig. 6A). The dependencies between these variables are expressed by arrows in the network indicating the relation between any variable *X*_*i*_ (such as *R*_*j*_ or *U*_*k*_) and its direct causal antecedents denoted as *parents* (*X*_*i*_). Thus, depending on a particular network structure *S* with model parameters *μ*_*S*_ the joint probability distribution $P(\vec{X})=P(X_{1},X_{2},...,X_{N+M})$ can be split up into a product of conditional probabilities: $P(\vec{X}|S,\mu_{S})=\prod_{i=1}^{N+M}P(X_{i}|\text{parents}(X_{i}),S,\mu_{S})$. The structure *S* of the network determines the dependencies between the variables — that is the presence or absence of arrows — while the probabilities that specify the actual dependencies quantitatively are parameters of that structure. Therefore, ‘structural learning’ refers to learning the topology of such a network, whereas ‘parametric learning’ involves determining quantitatively the causal connections given by the structure. In particular, the problem of structural learning is exacerbated in the presence of hidden variables, because the hidden variables and the structure between them and the observables have to be inferred. This is the standard case in sensorimotor learning. For instance, in the rotation experiments described above the hidden variable is the rotation angle. If the nervous system can extract this hidden variable, the joint probability distribution over the sensorimotor space can be efficiently computed as $P(\vec{R},\vec{U})=P(\vec{U}|\mu_{S})P(\mu_{S}|\vec{R})$, where *μ* represents a rotation-specific hidden variable (Fig. 6B). Formally, the inference process during structural learning is split up into two steps: (a) computing the posterior probability *P*(*S*|*X*) of a certain structural model *S* given the data *X*, and (b) computing the posterior probability *P*(*μ*_*S*_|*S*, *X*) of the parameter *μ*_*S*_ given the structural model *S* and the data *X*. By using this formalism the concept of structural learning can be easily incorporated within the framework of Bayesian sensorimotor integration [97,98]. What is not shown explicitly in Fig. 6A and B is the time-dependence of the random variables $\vec{R}$ and $\vec{U}$. However, time can be included by extending the graph to a Bayesian Network that represents sequences of these random variables. This is called a Dynamic Bayesian Network or DBN [99]. It is also straightforward to use structure learning in Bayesian nets as a modelling tool to understand transfer between different tasks [100].

## 6 Conclusions

The hypothesis of structural learning as a ‘learning to learn’ mechanism can be applied to a large body of existing research. In experimental psychology ‘learning to learn’ phenomena have been reported when animals are exposed to different environments that belong to a particular class or type. Instead of talking about ‘class’ or ‘type’ we might equally say that these environments share a common structure. Harlow pioneered the hypothesis that animals form ‘learning sets’ of abstract solution strategies applicable to all environments that share the same class or structure. In addition, studies in cognitive science have investigated structure learning mainly in the context of inferring causal dependencies. So far these studies have rarely asked the question how such structure learning transfers to new learning problems, although some have reported ‘learning to learn’ effects [39]. Thus, these lines of research provide evidence for structural learning in both animal and human cognitive learning tasks. The applicability of the concept, however, stretches much farther and includes, for example, even ‘learning to learn’ on an evolutionary scale where our ontogenetic learning is merely an adaptation to a particular instantiation of a living environment for which we have been selected [101]. Here, we have mainly focused on structure learning in motor control tasks. These findings are particularly interesting because previously it has often been thought that learning in highly variable environments cannot be achieved or that only average responses could be learned [102–107]. Recent data indicates, however, that this is not always the case and that structure learning can extract more abstract invariants. Structural learning in the motor system would then imply the learning of abstract motor strategies that are applicable in a wide range of environments that share common structures. Such an ability for primitive, non-cognitive abstraction or motor concept formation might also provide an interesting link between motor control and cognitive science [108]. In summary, structural learning might not only play a fundamental role in skill learning and underlie the unsurpassed flexibility and adaptability of the motor system, but also govern important cognitive learning processes observed in animal psychology and cognitive science.
